# Effectiveness and Usability of a Novel Kinect-Based Tailored Interactive Fall Intervention System for Fall Prevention in Older People: A Preliminary Study

**DOI:** 10.3389/fpubh.2022.884551

**Published:** 2022-05-31

**Authors:** Taekyoung Kim, Shuping Xiong

**Affiliations:** Department of Industrial and Systems Engineering, College of Engineering, Korea Advanced Institute of Science and Technology (KAIST), Daejeon, South Korea

**Keywords:** aging, fall prevention, fall risk, risk assessment and intervention, effectiveness, usability, Kinect

## Abstract

Falls are prevalent among older people and can lead to serious health problems. We newly developed a novel Kinect-based tailored interactive fall intervention system, which seamlessly integrates multifactorial fall risk assessment and tailored intervention programs to prevent falls in older people. This preliminary study aimed to examine the effectiveness and usability of this developed system for fall prevention in older people. Thirty community-dwelling older women participated in this experiment; they were allocated to an intervention group (IG) or a control group (CG) for a quasi-randomized trial (15 people each). Participants in IG followed an 8-week tailored intervention (40 min/session × 2 sessions/week × 8 weeks) using the Kinect-based interactive fall intervention system, while participants in CG maintained their habitual activities. Various outcome measures were evaluated at baseline (Week 0), interim (Week 4), and post-intervention (Week 8). Experimental results showed that IG led to significant improvements in TUG-Timed Up and Go (*p* = 0.010), BBS-Berg Balance Scale (*p* = 0.011), and Montreal Cognitive Assessment-MoCA (*p* = 0.022) between baseline and post-intervention. In comparison to the baseline, TUG and BBS were even significantly improved at interim (*p* = 0.004 and 0.047, respectively). There were no significant changes in static balance-related performance outcomes and the Short Falls Efficacy Scale-SFES after the intervention. Whereas in CG, most performance measures did not show significant changes during the 8-week period, TUG completion time became significantly longer at post-intervention in comparison to interim (*p* = 0.028) and fear of falling was also significantly higher at post-intervention than baseline (*p* = 0.021). These findings suggest that the Kinect-based 8-week tailored interactive fall interventions effectively improved older people's physical and cognitive abilities. Regarding the usability of the developed system, the average System Usability Scale (SUS) score was 83.5 out of 100, indicating excellent system usability. The overall mean Computer Literacy Scale (CLS) score was 2.5 out of 26, showing that older participants in this study had very limited experience with computers. No significant correlation between SUS and CLS scores demonstrated that newly developed Kinect-based tailored interactive fall intervention system was easy to use for older people, regardless of their computer experience. This novel system should help health professionals and older people proactively manage the risk of falls.

## Introduction

Falls are prevalent among older people, with ~30% of community-dwelling older people aged 65 or over experiencing a fall each year (1). Falls are the leading cause of fatal injuries and emergency medical visits, requiring more than $30 billion in direct medical costs annually to treat (2). Therefore, it is important to develop fall intervention programs and technologies to reduce fall risks and prevent falls.

Physical exercise is a typical fall intervention program that has been shown to be effective in reducing fall risk (3). Nevertheless, multifactorial fall interventions are necessary to maximize intervention effectiveness because falls are caused by a complex interaction of multiple fall risk factors (4–6). The Matter of Balance is a representative fall intervention program with a multi-component approach. This program includes exercises for strength, balance, range of motion, cognitive restructuring, and education to address multiple fall risk factors (7). This kind of conventional fall intervention program is typically offered through direct supervision by professional therapists in formal rehabilitation centers and clinical settings. Conventional rehabilitation programs have been effective at preventing falls; however, they suffer from low program adherence since they are passive and difficult for the older people to sustain due to lack of sense of active involvement, cost-ineffectiveness, and low accessibility (8, 9). With the rapid development of sensing and digital technologies, different types of fall interventions have been developed, such as telehealth programs and exergames (10). Exergaming (exercise + gaming) is gaining popularity and appear promising over passive conventional fall interventions due to intervention effectiveness, fun and high program adherence, and time/resource efficiency (9, 11, 12). However, most exergames rely on off-the-shelf systems with commercial software (e.g., Kinect XBOX, Wii Fit) and they were initially intended for younger and healthier users, not specifically developed for the older people (9).

Several studies have developed Kinect or wearable sensor-based fall intervention programs for older adults, such as the Otago Exercise Program and Tai-Chi (13, 14). Chen et al. (15) developed Kinect-based exergames with multi-components to train lower-limb muscles, visuospatial ability, attention, and executive function. Through narrative interviews and user experience questionnaires, the researchers reported that older adults were impressed by the exergames and enjoyed playing them. Yu and Xiong (16) even added a virtual coach to their Tai-Chi exergames to support Kinect-based unsupervised home rehabilitation. Older users were able to imitate the Tai-Chi movements demonstrated by the virtual coach, and the system can continuously monitor the trainees' movements and provide real-time performance feedback.

Relatively few studies have attempted to develop customized exergame-based multifactorial fall intervention systems for older people and to examine the effectiveness of these systems in specific intervention periods. Gschwind et al. (11) developed iStoppFalls system using a Microsoft Kinect, a Senior Mobility Monitor (3D accelerometer and barometer), and a PC and TV set top box; the system provides exergames of balance and strength for older people. Once participants reached higher difficulty levels, cognitive tasks were added to balance exergames. Through the 16-week intervention, the intervention group performed significantly better than the control group in terms of Physiological Profile Assessment (PPA); in addition, in the dual task, the system effectively reduced the hand reaction time and completion time of the 10-m walk. The system, however, could be expensive and inconvenient for older people because it requires a Senior Mobility Monitor to be worn by user, aside from the Kinect. Martins et al. (17) implemented lower-limb exercises into a technical system using a single inertial sensor. They performed an 8-week intervention that showed significant improvements in hand grip strength, step test, and sit-to-stand. Ogawa et al. (12) also developed Kinect-based exergames to train balance and cognitive function. After 8 weeks of training, the intervention group significantly improved cognitive performance in the Mini-Mental State Examination and Trail Making Test, while the traditional exercise group significantly improved physical function. Even though these studies are valuable and show the effectiveness of exergame-based multifactorial fall interventions, most early studies have not seamlessly integrated their fall risk assessments and intervention programs for tailored and effective interventions (18). Therefore, older users are offered the same intervention programs at the outset, regardless of their individual fall risks and underlying risk factors. In addition, the difficulty level of the fall intervention program should be dynamically adjusted based on scientific principles and user performance for tailored intervention and continuous improvement (19), yet many studies do not explicitly address the level of difficulty (12, 13, 17, 20).

**Figure 1:**
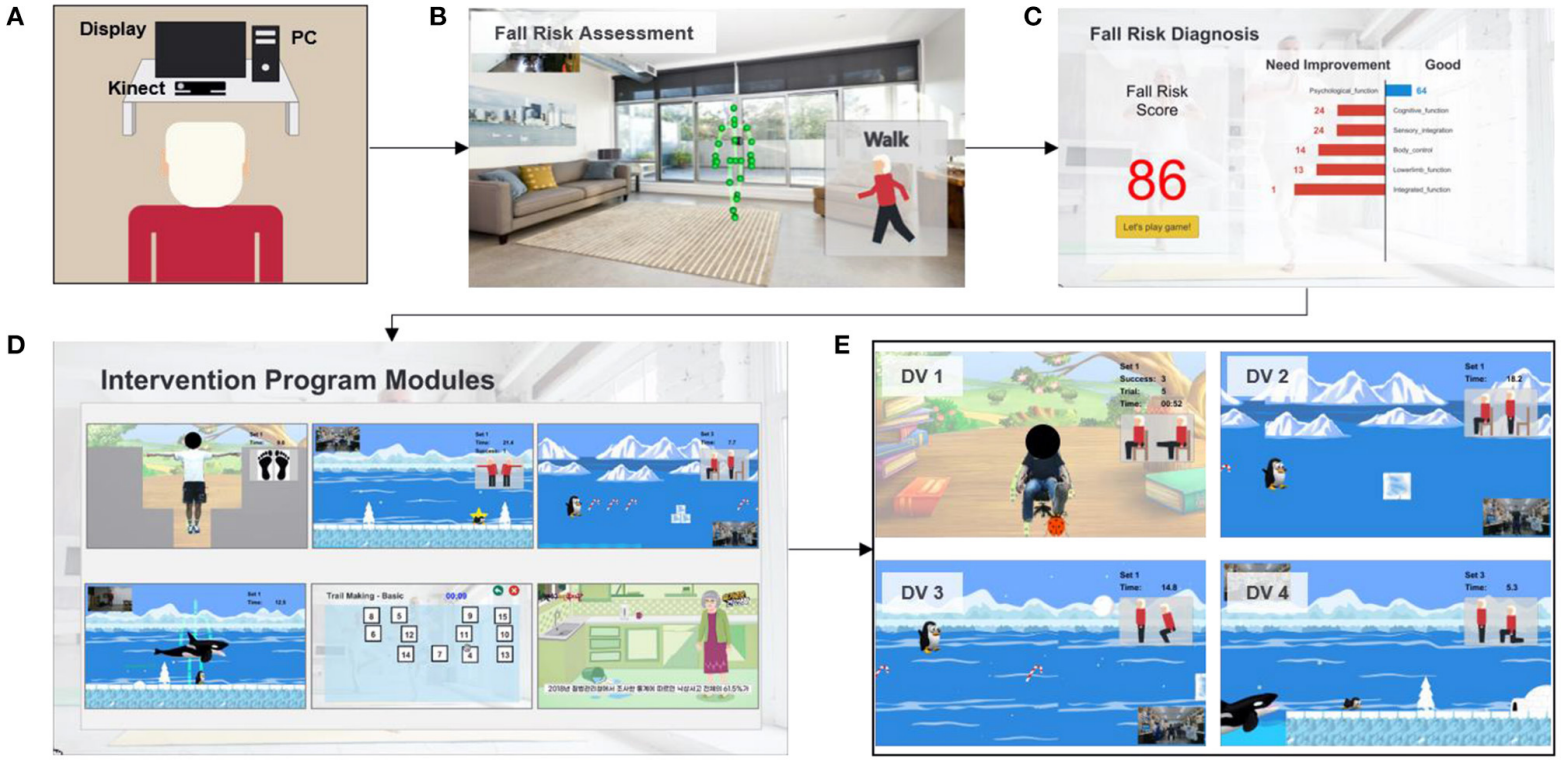
Overall workflow of a novel Kinect-based tailored interactive fall intervention system.

To overcome the limitations of existing studies, we developed a novel Kinect-based tailored interactive fall intervention system (Figure 1). The system was low-cost, markerless, and interactive because of the good features of Microsoft Kinect. The Microsoft Kinect is inexpensive ($249 for Kinect v2), markerless (unobtrusive), and capable of tracking full-body motion in real time. In addition, Kinect applies RGB-D sensors and embedded gesture recognition algorithms to infer the players' skeleton and movement of body segments, allowing the player to directly interact with the intervention program through gestures without having to hold any controllers. The developed system also seamlessly integrated multifactorial fall risk assessment and tailored intervention programs to prevent falls in older people. Figure 1A shows the system hardware setup, which consists of a Microsoft Kinect and a desktop computer placed on a table. The fall risk assessment included a Kinect-based practical test battery (21) to comprehensively evaluate major fall risk factors for physical, cognitive, and integrated functions (Figure 1B). The system can estimate an individual's overall fall risk based on the probability of falling *via* a machine learning-based fall risk classification model. An individual's performance on the test battery was computed as the percentile value of a normative database (22) so that deficiencies could be clearly visualized and targeted for tailored intervention (Figure 1C). Modular exergame programs (some for physical rehabilitation, some for cognitive training, etc.) with four difficulty levels were also developed to provide tailored fall interventions for older people (Figure 1D). The difficulty level of the program in each module was determined at the outset by individual fall risk assessment results; it was increased according to intervention progress to continuously offer challenging programs. The difficulty levels were adjusted based on scientific principles and input from health professionals. For example, difficulty levels (DV) of an intervention program for the lower-limb function were progressive based on the following principles: use more joints (DV 1 → DV 2) and reduce the base of support (DV 2 → DV 3 or DV 3 → DV 4) as shown in Figure 1E. In addition, quantitative progressions were used to make exergames harder, including changes in speed, performance time, and number of repetitions. Older individuals performed the tailored intervention programs, and if their performance was insufficient, the difficulty level of the program would not change. If older people completed the program successfully, the difficulty level would dynamically increase. A detailed description of the entire system is reported elsewhere and is beyond the scope of this study.

This preliminary study aimed to examine the effectiveness and usability of the newly developed Kinect-based tailored interactive fall intervention system for fall prevention in older people.

## Methods

### Study Design and Participants

Thirty community-dwelling older people were recruited from a senior welfare center in the metropolitan city of Daejeo, South Korea. The inclusion criteria were as follows: aged 65 or older, female, and able to walk independently without the use of assistive devices. Only females were recruited in this study to avoid the influence of gender differences on fall risk, as older females were reported to have a higher risk of falls than males (21, 23). To minimize the confounding effect of age on fall risk, the recruited participants were allocated to an intervention group (IG) or a control group (CG) of 15 people each *via* a quasi-randomized trial (24). Each participant provided signed informed consent prior to participation. Their self-reported fall history in the past 1 year was also collected. This study was ethically approved by the KAIST Institutional Review Board (IRB No: KH2021-194).

**Figure 2:**
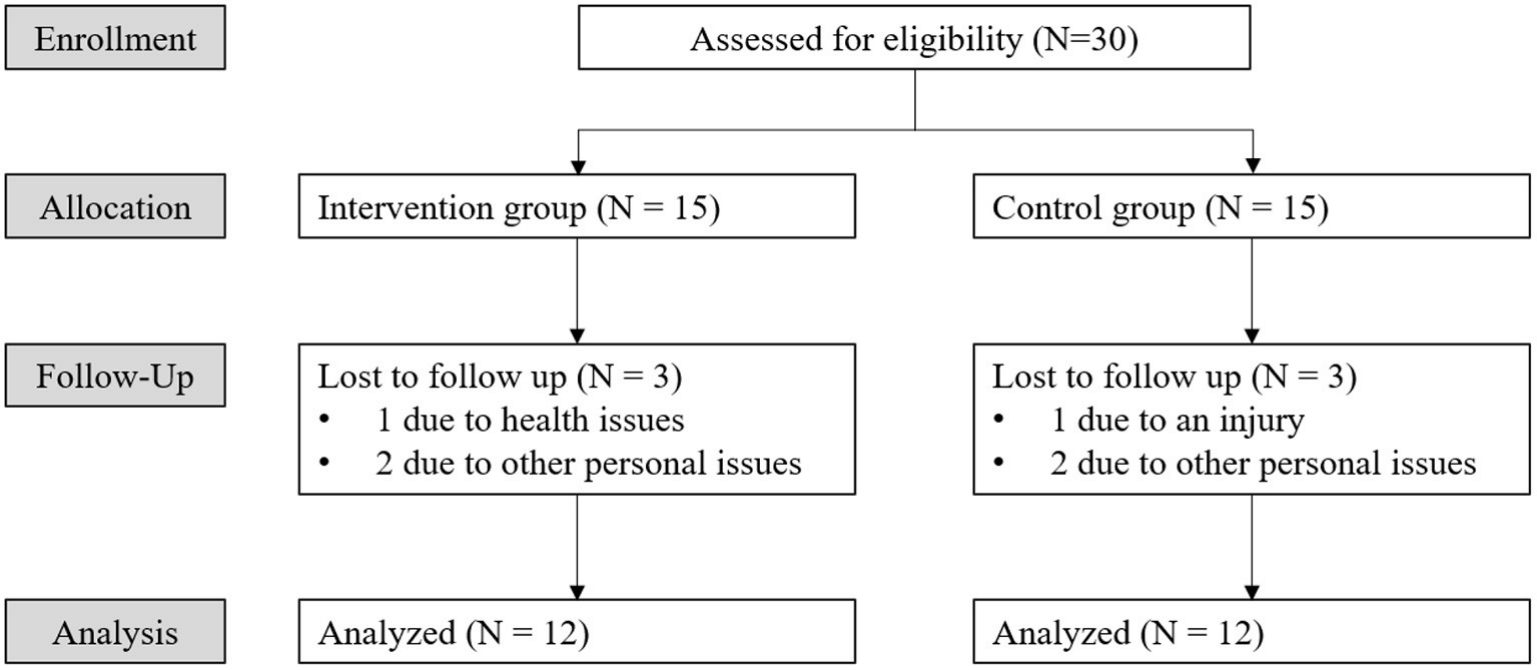
Consolidated standards of reporting clinical Trial (CONSORT) flowchart representing status of participants through the 8-week study.

Throughout the 8-week experiment, 3 participants each in IG and CG withdrew due to health issues or personal issues (Figure 2), and 12 (=15–3) participants in each group successfully completed (Table 1). Table 1 summarizes the demographic information of both groups. IG was significantly older (*p* = 0.035) and shorter (*p* = 0.018) than CG. At the beginning, the age difference between IG and CG was not statistically significant (IG: 77.6 ± 6.3; CG: 74.6 ± 4.8; *p* = 0.546); however, it became significant after the dropouts. We found that, in general, relatively young older women in IG tended to have active personal schedules, making it more difficult to regularly complete the 8-week intervention program (two sessions per week). Some of them also believed that the intervention program was mainly for frail older adults or patients. Therefore, they dropped out of the regular intervention program, resulting in participants in IG being older and shorter than CG, because height becomes shorter as age advances in the older population (25).

**Table 1:** Demographic characteristics (mean, standard deviation in bracket) of the intervention group and the control group.

**Characteristics**	**Intervention group, IG (*N* = 12)**	**Control group, CG (*N* = 12)**	**Two-sample comparison (*p*-value)**
Age (year)	78.67 (5.91)	73.75 (4.75)	0.035^*^
Height (cm)	151.55 (4.36)	156.67 (5.41)	0.018^*^
Weight (kg)	55.53 (6.50)	55.84 (8.32)	0.918
BMI (kg/m^2^)	24.17 (2.64)	22.77 (3.27)	0.261
Number of falls in the past 1 year	0.25 (0.45)	0.50 (0.67)	0.299

* *Significant difference with p < 0.05*.

### Interventions

Participants in IG followed an 8-week tailored intervention (40 min/session × 2 sessions/week × 8 weeks) that was recommended and implemented by the Kinect-based tailored interactive fall intervention system, while participants in CG maintained their habitual activities. Intervention sessions were initially conducted under the guidance of an experienced researcher but, over time, participants in IG performed the intervention programs more independently.

The intervention programs of the Kinect-based system were composed of six modules (Table 2): static balance, postural stability, lower-limb function, mobility, cognitive function, and fall education. Each module, except fall education, included four difficulty levels. Difficulty levels were determined at the outset by results of the Kinect-based fall risk assessment and increased according to individual performance improvements while performing intervention programs.

**Table 2:** Kinect-based modular fall intervention programs and description.

**Intervention module**	**Graphic illustration**	**Descriptions**
Static balance	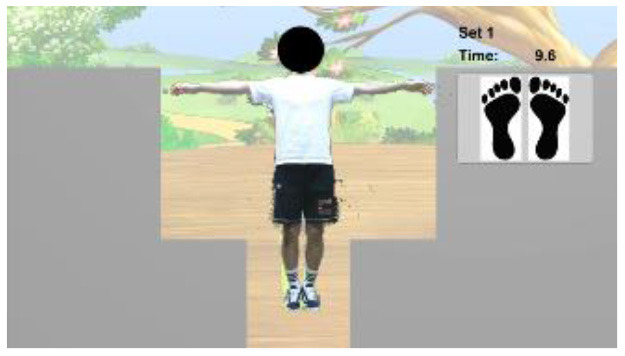	Level 1: Standing with feet together Level 2: Semi-tandem standing Level 3: Tandem standing Level 4: One leg standing A participant should maintain static balance for 30 s with the required stance in each level Each program is composed of 3 sets. If a participant successfully completes 3 sets, the difficulty level is progressed
Postural stability	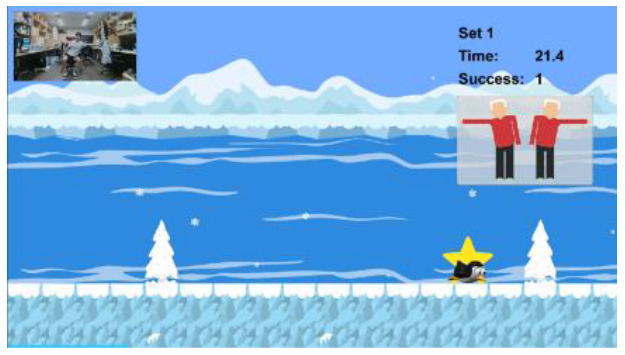	Level 1: Sit on the chair and reach Level 2: Advanced sit on the chair and reach Level 3: Stand and reach Level 4: Advanced stand and reach A participant sits on the chair (or stands) and should touch an asterisk by reaching left or right depending on where the asterisk appears Each program is composed of 3 sets. If a participant successfully completes 3 sets, the difficulty level is progressed
Lower-Limb function	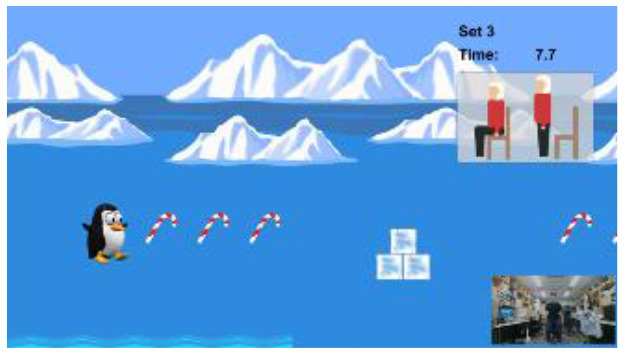	Level 1: Knee extension sitting on the chair Level 2: Sit-to-stand on the chair Level 3: Squat Level 4: Lunge A participant should perform the required exercise for 30 s in each level to keep a penguin on the screen flying to the destination, not to sink under the sea Each program is composed of 3 sets. If a participant successfully completes 3 sets, the difficulty level is progressed
Mobility	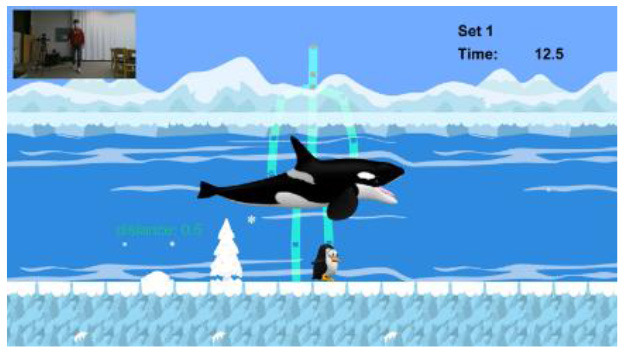	Level 1: Hip flexion sitting on the chair Level 2: Walking in place Level 3: Walking back and forth Level 4: Walking back and forth with changed speed A participant should perform the required task for 30 s in each level to make a penguin maintain a certain distance from a moving whale Each program is composed of 3 sets. If a participant successfully completes 3 sets, the difficulty level is progressed
Cognitive function	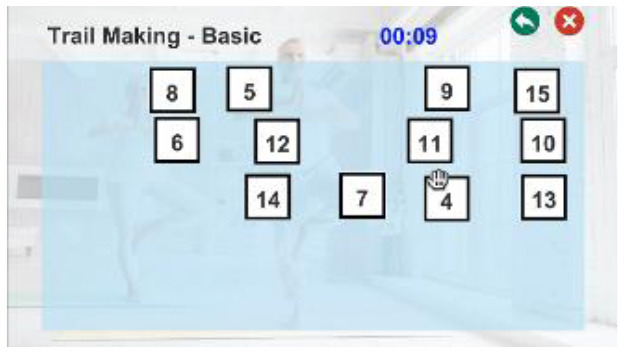	Level 1: Forward trail making Level 2: Backward trail making Level 3: Trail making with two colors Level 4: Trail making for numbers & figures A participant should select 15 cards in order correctly by griping and releasing the hand If a participant successfully completes a program within the threshold time and errors, the difficulty level is progressed.
Fall education	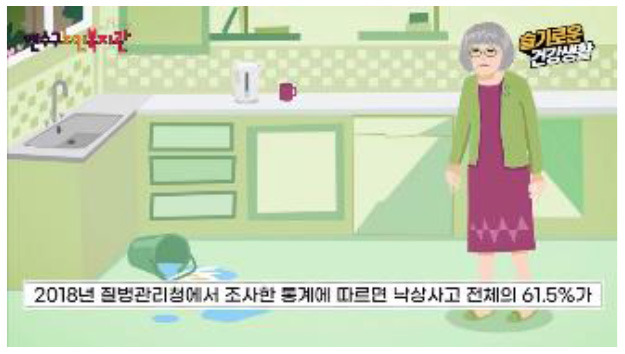	Education 1: Regulations to prevent falls Education 2: Nutrition and fall prevention A participant should watch the 10-min educational videos to learn why falls happen, how to prevent them, and how to have a better health condition in daily living

### Outcome Measures

To evaluate the effectiveness of the intervention, various performance outcomes of both IG and CG were measured at baseline (Week 0: before intervention), interim (Week 4: during intervention), and post-intervention (Week 8: after intervention) using established fall risk assessment tools.

**Figure 3:**
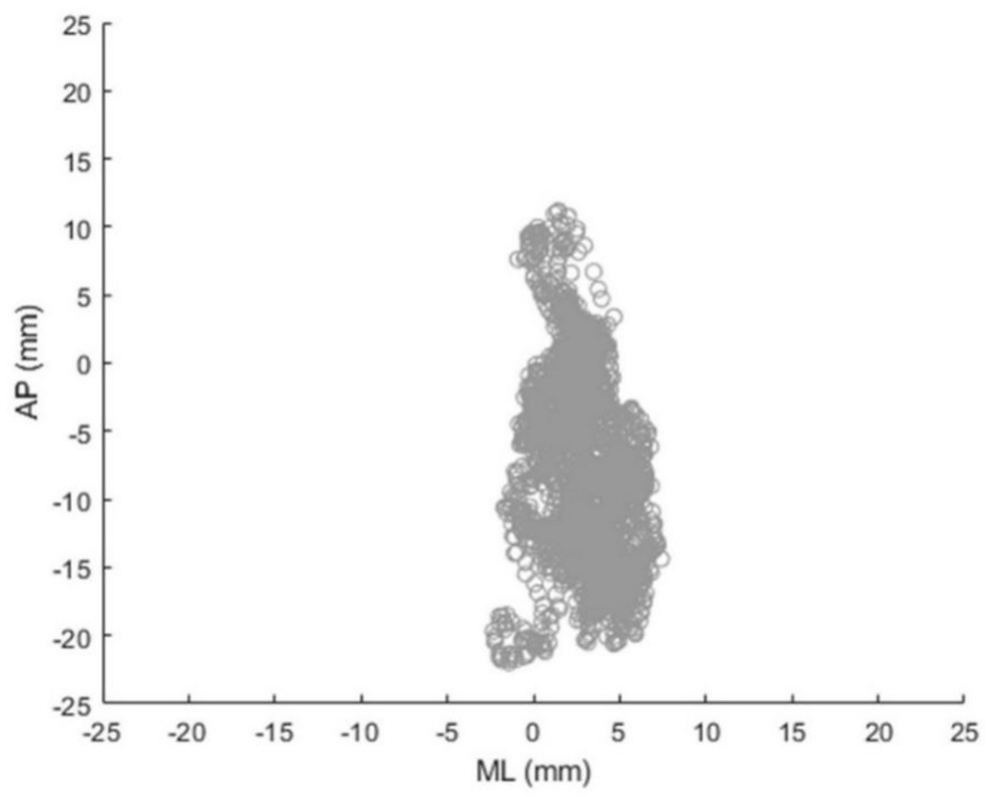
Exemplary center-of-pressure (CoP) trajectory during static standing measured by Nintendo Wii Balance Board (AP, anterior-posterior; ML, medio-lateral).

To assess static balance, body sway during static standing with eyes open and closed was quantified with a Nintendo Wii Balance Board (26, 27). Sway ranges and sway velocities in anterior-posterior (AP) and medio-lateral (ML) directions were calculated from center-of-pressure (CoP) trajectories (Figure 3), (28). Timed Up and Go (TUG) was conducted to measure functional mobility and Berg Balance Scale (BBS) was used to assess balance ability. TUG is a tool that assesses mobility and integrated function; it involves standing from a chair, walking 3 m at normal pace, turning, walking back, and sitting on the chair. The outcome measure of TUG is the total completion time; a longer time indicates poorer functional mobility and higher fall risk (29). BBS is composed of 14 items that assess balance ability. Each item can be scored from 0 to 4; therefore, the maximum score is 56, with a lower score indicating poorer balance and higher fall risk (30, 31). Montreal Cognitive Assessment (MoCA) was performed to evaluate cognitive performance including executive function and attention. MoCA is the 30-point cognitive screening test for people with mild cognitive impairment; a lower score indicates poorer cognitive performance (32). A shortened version of the Fall Efficacy Scale (SFES) was used to assess the fear of falling. SFES is a 7-item scale to measure fear of falling. Each item can be scored 1–4 based on the level of concern about falling for the given daily activities. The score range is 7–28; a higher score indicates higher fear of falling (33). One research assistant was always standing by to ensure the safety of the experimental participants.

**Figure 4:**
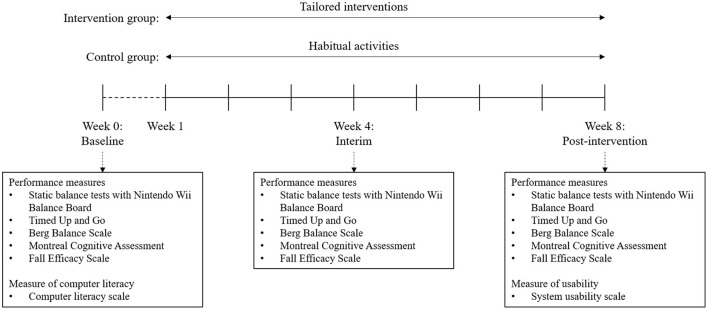
Study design for the 8-week intervention and the outcome measures at each assessment moment (baseline, interim, and post-intervention).

The usability of the Kinect-based tailored interactive fall intervention system was investigated by the System Usability Scale (SUS) at post-intervention. SUS is widely used to measure system usability, and previous studies have reported that it is reliable and valid to evaluate learnability as well as usability (34). It consists of 10 items, each rated on a 5-point Likert scale ranging from 1-strongly disagree to 5-strongly agree. Each participant's computer experience was also examined using the Computer Literacy Scale (CLS), as it may affect the usability of this newly developed system. CLS is an objective knowledge test to assess basic understanding of symbols and terms in the user interface of interactive computer technology (35). People who were familiar with computers are generally more favorably disposed to ICT-based systems. All utilized outcome measures at each assessment are presented in Figure 4.

### Statistical Analysis

Descriptive statistics (mean, standard deviation) were used to summarize the experimental data. The assumptions of parametric statistical analysis, including normality of data, homogeneity of variance, and sphericity, were first checked by Shapiro-Wilk, Levene, and Mauchly tests, respectively. Since some outcome measures did not follow normal distributions and the sample size of this preliminary study was relatively small, non-parametric tests were used for all statistical analyses (36) except two-way mixed analysis of variance (ANOVA), as there was no suitable non-parametric alternative to two-way mixed ANOVA.

Two-way mixed analysis of variance (ANOVA) with intervention period as the within-subjects factor and group as the between-subjects factor was carried out to investigate the effects of group (IG, CG), intervention period (baseline, interim, post-intervention), and their potential interaction effect (group × period). The Greenhouse-Geisser correction was applied if sphericity was violated. Two separate Friedman tests and *post-hoc* analyses using Wilcoxon signed rank tests were performed for both groups (IG, CG) to examine the effect of intervention period more specifically. In addition, as a secondary analysis, if the effect of intervention period was found to be statistically significant for some outcome measure, the pre-post changes over the 8-week period for both groups in that outcome measure were also calculated to examine any group difference by using the Mann-Whitney U test. In addition to statistical significance, effect sizes were further estimated to check practical significance. For two-way mixed ANOVA, partial eta squared ($\eta_{\text{p}}^{2}$) was used to estimate the effect size and a basic rule on the magnitudes of the effect size is as follows (37, 38): $\eta_{\text{p}}^{2}$ ~ 0.01 (small), $\eta_{\text{p}}^{2}$ ~ 0.06 (medium), and $\eta_{\text{p}}^{2}$ ~ 0.14 (large). For Friedman test, Kendall's W was used to estimate the effect size by the following criteria (39, 40): W ~ 0.1 (small), W ~ 0.3 (medium), and W ~ 0.5 (large). For Mann-Whitney *U*-test and Wilcoxon signed rank test, effect size r (=Z/$\sqrt{N}$) proposed by Cohen was used to estimate the effect size as follows (37, 38): effect size *r* ~ 0.1 (small), effect size *r* ~ 0.3 (medium), and effect size *r* ~ 0.5 (large).

All statistical analyses were performed using IBM SPSS Statistics 20 (IBM Corporation, New York, United States) with a significance level of 0.05.

## Results

### System Effectiveness

The results of the mixed ANOVA (2 groups × 3 intervention periods) are shown in Table 3 and data for all performance outcomes are summarized in Table 4. There was no significant main effect of group for any outcome measure, indicating no overall difference between the IG and CG groups. The main effect of intervention period was significant in TUG [*F*_(2,44)_ = 5.98; *p* = 0.005; $\eta_{\text{p}}^{2}$ = 0.214], BBS [*F*_(2,44)_ = 6.24; *p* = 0.004; $\eta_{\text{p}}^{2}$ = 0.221], and MoCA [*F*_(1.5,34.1)_ = 4.89; *p* = 0.020; $\eta_{\text{p}}^{2}$ = 0.182], indicating that the participants exhibited different TUG, BBS and MoCA outcome performances after the intervention. However, there were no significant changes in static balance measures (sway range and sway velocity) or SFES after the intervention. Regarding the interaction effect, there were no significant interactions between group and intervention period for any of the outcome measures except TUG completion time [*F*_(2,44)_ = 10.45; *p* < 0.001; $\eta_{\text{p}}^{2}$ = 0.322] and BBS score [*F*_(2,44)_ = 2.94; *p* = 0.063; $\eta_{\text{p}}^{2}$ = 0.118]. The TUG completion time for IG was significantly shorter at post-intervention compared to the baseline; however, it became significantly longer in CG during the same period. The interaction effect of “group × intervention period” on BBS score was marginally significant (0.05 < *p* < 0.10): the BBS score of IG was significantly increased after intervention compared to baseline; however, CG remained almost unchanged over the same period (Table 4).

**Table 3:** Mixed ANOVA results for outcome measures.

**Outcome measures**		**Factor effect**	***F*-ratio**	***p*-value**	** $\eta_{p}^{2}$ **
TUG completion time (sec)		Group	3.648	0.069^‡^	0.142
		Period	5.975	0.005^*^	0.214
		Group × period	10.451	<0.001^*^	0.322
BBS score		Group	0.307	0.585	0.014
		Period	6.243	0.004^*^	0.221
		Group × period	2.938	0.063^‡^	0.118
MoCA score ^#^		Group	2.034	0.168	0.085
		Period	4.888	0.020^*^	0.182
		Group × period	0.965	0.371	0.042
SFES score		Group	0.645	0.430	0.029
		Period	2.300	0.112	0.095
		Group × period	1.131	0.332	0.049
Static balance measures	SR (AP, EO)^#^ (mm)	Group	0.170	0.684	0.008
		Period	0.859	0.401	0.038
		Group × period	0.533	0.537	0.024
	SR (AP, EC) (mm)	Group	3.344	0.081^‡^	0.132
		Period	1.500	0.234	0.064
		Group × period	0.631	0.537	0.028
	SR (ML, EO) (mm)	Group	0.556	0.464	0.025
		Period	0.638	0.533	0.028
		Group × period	0.959	0.391	0.042
	SR (ML, EC) (mm)	Group	0.462	0.504	0.021
		Period	0.143	0.867	0.006
		Group × period	0.259	0.773	0.012
	SV (AP, EO)^#^ (mm/s)	Group	0.000	0.983	0.000
		Period	0.065	0.886	0.003
		Group × period	0.493	0.559	0.022
	SV (AP, EC) (mm/s)	Group	0.005	0.945	0.000
		Period	0.204	0.816	0.009
		Group × period	2.283	0.114	0.094
	SV (ML, EO)^#^ (mm/s)	Group	0.019	0.891	0.001
		Period	1.071	0.336	0.046
		Group × period	0.553	0.530	0.025
	SV (ML, EC) (mm/s)	Group	0.022	0.883	0.001
		Period	1.207	0.309	0.052
		Group × period	0.422	0.658	0.019

* *Significant difference with p < 0.05*;

‡ *Marginal significance with 0.05 < p < 0.10*.

# *Greenhouse-Geisser correction was applied if the assumption of sphericity was violated*.

*BBS, berg balance scale; MoCA, montreal cognitive assessment; SFES, shortened version of the fall efficacy scale; TUG, timed up and go; AP, anterior-posterior; ML, medio-lateral; EC, eyes closed; EO, eyes open; SR, sway range; SV, sway velocity*.

**Table 4:** Performance outcomes (mean, standard deviation in bracket) of the intervention group and the control group at baseline, interim, and post-intervention, and results of Friedman tests and *post-hoc* analyses.

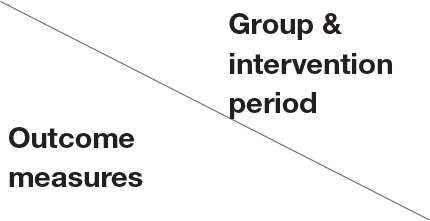		**Intervention group, IG (*****N*** **=** **12)**				**Control group, CG (*****N*** **=** **12)**			
		**Baseline (Week 0)**	**Interim (Week 4)**	**Post-intervention (Week 8)**	**Kendall's W**	**Baseline (Week 0)**	**Interim (Week 4)**	**Post-intervention (Week 8)**	**Kendall's W**
TUG completion time (sec)		12.4 (3.6)	11.2 (3.3)^a^	11.4 (2.9)^b^	0.583	9.5 (1.5)	9.5 (1.7)	10.0 (1.4)^c^	0.140
BBS score		51.3 (4.8)	53.2 (3.3)^a^	53.7 (2.8)^b^	0.360	53.1 (2.5)	53.7 (2.4)	53.4 (2.5)	0.081
MoCA score		18.8 (6.2)	20.7 (6.8)	21.2 (4.9)^b^	0.195	22.9 (4.4)	23.6 (5.9)	23.8 (5.9)	0.072
SFES score		14.3 (4.5)	14.7 (4.5)	14.6 (4.2)	0.143	12.0 (3.4)	13.3 (4.6)	14.1 (5.4)^b^	0.161
Static balance measures	SR (AP; EO) (mm)	30.0 (21.1)	28.3 (7.8)	25.6 (7.6)	0.090	32.0 (14.7)	26.9 (8.0)	30.1 (12.3)	0.063
	SR (AP; EC) (mm)	33.1 (19.0)	29.5 (11.0)	29.8 (6.6)	0.049	40.5 (10.8)	39.8 (14.9)	33.4 (10.6)	0.188
	SR (ML; EO) (mm)	20.6 (9.7)	20.6 (8.2)	19.9 (10.7)	0.028	16.3 (6.5)	19.3 (7.1)	18.8 (4.9)	0.132
	SR (ML; EC) (mm)	22.6 (16.2)	24.6 (19.7)	20.8 (9.2)	0.028	21.3 (6.7)	19.7 (6.1)	20.3 (7.7)	0.028
	SV (AP; EO) (mm/s)	12.0 (4.3)	11.6 (1.9)	11.5 (2.5)	0.083	11.6 (2.1)	11.8 (2.1)	11.8 (2.1)	0.007
	SV (AP; EC) (mm/s)	17.1 (9.0)	15.9 (5.5)	16.0 (5.1)	0.007	15.2 (3.8)	17.5 (4.8)	16.7 (5.0)	0.174
	SV (ML; EO) (mm/s)	12.3 (2.5)	12.5 (2.5)	12.0 (1.9)	0.111	12.2 (2.4)	12.6 (2.5)	12.4 (2.3)	0.174
	SV (ML; EC) (mm/s)	13.3 (2.9)	13.1 (3.0)	12.5 (1.9)	0.090	12.8 (2.9)	12.9 (2.8)	12.7 (2.4)	0.028

a *Significant difference between baseline and interim*.

b *Significant difference between baseline and post-intervention*.

c *Significant difference between interim and post-intervention; BBS, berg balance scale; MoCA, montreal cognitive assessment; SFES, shortened version of the fall efficacy scale; TUG, timed up and go; AP, anterior-posterior; ML, medio-lateral; EC, eyes closed; EO, eyes open; SR, sway range; SV, sway velocity*.

**Figure 5:**
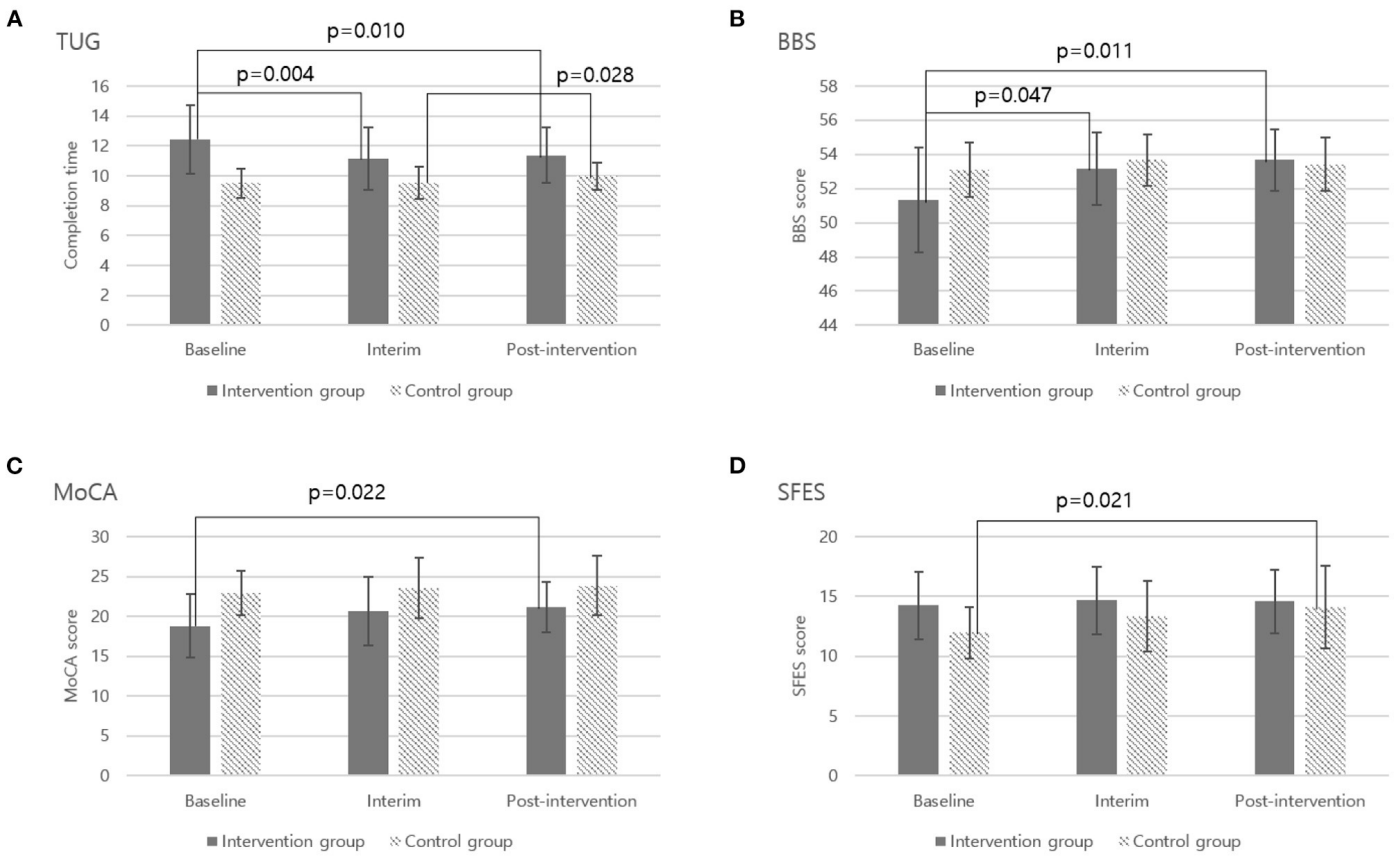
Significant outcome measures from two separate Friedman tests and *post-hoc* analyses. **(A)** Timed Up and Go-TUG, **(B)** Berg Balance Scale-BBS, **(C)** Montreal Cognitive Assessment-MoCA, **(D)** Shorten Fall Efficacy Scale-SFES.

Table 4 shows the results of the two separate Friedman tests and *post-hoc* analyses for performance outcomes of the IG and CG at baseline, interim and post-intervention. IG had significant improvements in TUG (*p* = 0.010; effect size *r* = 0.748), BBS (*p* = 0.011; effect size *r* = 0.731), and MoCA (*p* = 0.022; effect size *r* = 0.663) between baseline and post-intervention. Figure 5 provides details of these significant results. The TUG completion time of IG at post-intervention (11.4 s) was significantly shorter than the baseline (12.4 s) by 8% (Figure 5A). TUG of IG even significantly improved at interim by comparison to baseline (11.2 vs. 12.4 s; *p* = 0.004; effect size *r* = 0.838). The mean BBS score for IG at baseline was 51.3; this increased significantly by 5% to 53.7 at post-intervention (Figure 5B), and this measure was also significantly improved at interim in comparison to baseline (53.2 vs. 51.3; *p* = 0.047; effect size *r* = 0.574). Likewise, the mean MoCA score for IG was 18.8 at baseline and increased significantly by 12% to 21.2 at post-intervention (Figure 5C).

Whereas in CG, most performance measures did not show significant changes during the 8-week period. However, the TUG completion time became significantly longer by 5% at post-intervention in comparison to interim (9.5 vs. 10.0 s, *p* = 0.028; effect size *r* = 0.634; Figure 5A) and fear of falling was also significantly higher by 17% at post-intervention than baseline (12.0 vs. 14.1, *p* = 0.021; effect size *r* = 0.665; Figure 5D).

Table 5 further illustrates the detailed pre-post changes (i.e., the performance improvements) in performance outcomes for the IG and CG after the 8-week intervention. Overall, IG showed better performance improvements than CG in TUG, BBS, MoCA, and SFES, and the performance improvements in TUG (*p* < 0.001; effect size *r* = 0.672) and BBS (*p* = 0.060; effect size *r* = 0.401) were found to be significant and marginally significant, respectively.

**Table 5:** Comparison of pre-post changes in outcome measures between intervention and control groups (mean, standard deviation in bracket) after the 8-week intervention.

**Outcome measure**	**Pre-post change after the 8-week intervention (post-intervention-baseline)**		**Two-sample comparison**		
	**Intervention group, IG (*N* = 12)**	**Control group, CG (*N* = 12)**	**Mann–whitney *U***	***p*-value**	**Effect size r**
TUG completion time (sec)	−1.03 (1.06)	0.47 (0.94)	15.0	<0.001^*^	0.672
BBS score	2.33 (2.90)	0.33 (1.50)	39.0	0.060^‡^	0.401
MoCA score	2.33 (2.96)	0.92 (2.11)	53.0	0.291	0.226
SFES score	0.33 (2.61)	2.08 (2.53)	48.0	0.178	0.286

* *Significant difference with p < 0.05*;

‡ *Marginal significance with 0.05 < p < 0.10*.

### System Usability

**Figure 6:**
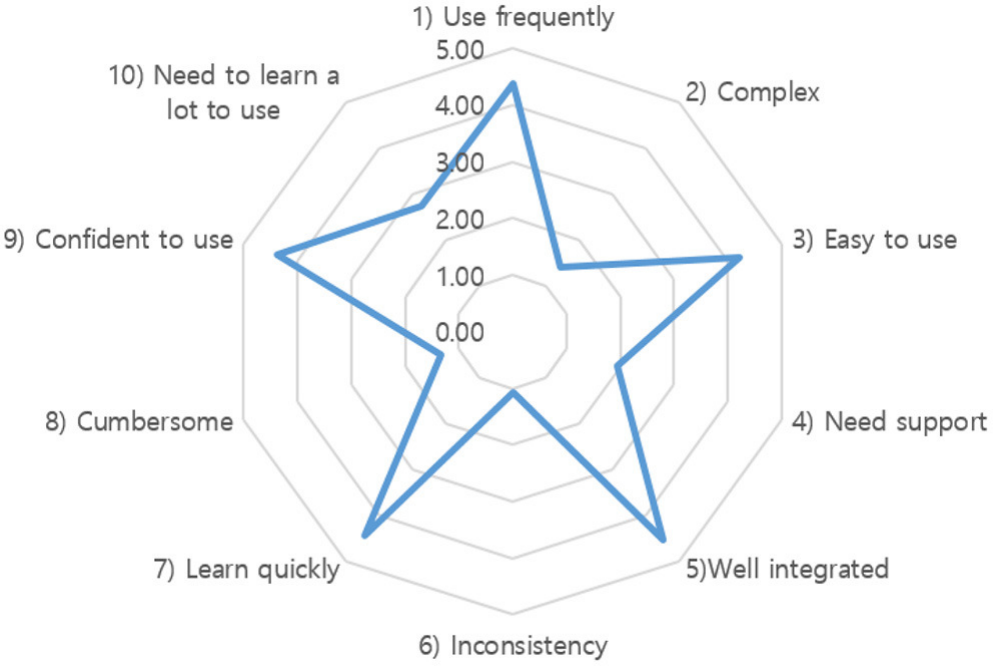
Results of the mean score of each assessment item in system usability scale (SUS).

The average SUS score was 83.5 out of 100, indicating excellent system usability (34, 41). Figure 6 provides details of all 10 assessment items. All the odd items had scores of over 4 (maximum 5), and all the even items had scores of <2, except for the 10th item (“need to learn a lot to use”), which was 2.75. Therefore, the overall subjective rating of the system was “easy to use,” “not complex,” “well-integrated,” “consistent,” and “able to learn quickly;” however, at the same time, participants felt that they needed some time to learn to use the system. In addition, the overall mean CLS score was 2.5 out of 26, showing that older participants in this study had very limited experience with computers. There was no significant correlation (*R* = 0.035, *p* = 0.870) between SUS and CLS scores, demonstrating that the developed Kinect-based tailored interactive fall intervention system was easy to use for older people, regardless of their computer experience.

## Discussion

This study investigated the effectiveness of our newly developed Kinect-based tailored interactive fall intervention system through an 8-week intervention. The program was found to have a beneficial effect on functional mobility (TUG), balance ability (BBS), and cognitive function (MoCA). Fear of falling (SFES) did not change significantly in IG, but fear of falling in CG became significantly higher; this implies that the 8-week fall intervention had a positive effect on relieving older people's concerns about falling.

The beneficial effects of the 8-week Kinect-based interactive fall intervention program for the older people were expected and largely consistent with previous studies. Improvements in physical and cognitive abilities were likely due to multifactorial and tailored fall interventions (4, 5, 7) and active engagement in video game-based interventions (9, 42). We carefully designed a multifactorial fall intervention with 6 modules covering static balance, postural stability, lower-limb function, mobility, cognitive function, and fall education, which were strongly associated with reducing risk of falls (7, 43). It is critical to include representative and valid fall intervention modules in the system, as the effectiveness of fall intervention is highly dependent on underlying fall risk factors, which may vary among different older adults (44). As shown in Table 2, we attempted to include representative intervention programs validated by previous studies. For example, intervention programs for static balance, lower-limb function, and mobility are widely used to improve older people's function (11, 17, 45). The Trail Making tests for cognitive training in this system are often used to assess cognitive function, but at the same time, they can be used as the intervention program because they train attention, sequencing, and cognitive flexibility (46). Furthermore, our system not only provided intervention programs with customized difficulty levels based on the results of the embedded multifactorial fall risk assessment, but also adjusted difficulty levels dynamically with scientific principles depending on user performance. For example, the module of lower-limb function increased difficulty levels by involving more joints, reducing BOS, and making the center of gravity higher. The module of cognitive function involved more advanced sequencing function and executive function as difficulty levels increased. Another important advantage of our Kinect-based fall intervention system is that it does not require additional physical devices (e.g., wearable sensor, balance board) to perform intervention programs compared to other exergame systems. This feature can minimize inconvenience and discomfort of older users, thereby enhancing their active participation in video game-based interventions. Interestingly, even though the intervention group's fear of falling didn't reduce significantly after the 8 week intervention, the control group's fear of falling was significantly increased after 8 weeks. This result was largely consistent with the study of Delbaere et al., although they conducted a slightly longer study of 3 months (47). There are two potential reasons for this result. First, the fear of falling could lead to stiffening strategies in behaviors that increase the fear of falling again (48). CG participants likely suffered more from this negative cycle by maintaining their habitual activities over the 8 week period, whereas IG participants actively received effective interventions to improve their functioning. Second, for CG participants at high fall risks, the biased attention (49) to fall risks may have made them pay more attention to fall risk, leading to a higher subjective fear of falling in SFES.

Surprisingly, even though our intervention system and 8 week intervention program resulted in a significant improvement in overall balance ability reflected in the BBS score, it did not have any significant effect on CoP-based static balance measures (Table 4). There are two potential reasons for this puzzling phenomenon. First, our developed fall intervention program and system was more effective for dynamic balance than static balance, so overall balance (BBS) was improved even when static balance remained unchanged. Second, the 8 week intervention period may not be sufficient to effectively train static balance. Despite conflicting results regarding the effects of fall intervention programs on static balance, we found that earlier studies reporting significant effects on static balance (50–52) trained their participants for at least 12 weeks, whereas our study provided only 8 weeks of intervention. Of course, different approaches to assessing the static balance between different studies may also lead to mixed results.

We investigated not only the effectiveness of the system for fall interventions, but also its usability for older people. The SUS score of our system was evaluated at 83.5, which suggests excellent usability in general (34). The usability of our system was rated higher or at least similar compared to previous studies using the SUS scale for their self-developed fall intervention systems (42, 53). We believe our system hardware setup, software features, and user interface all contribute to excellent system usability. Our system components included only a markerless Microsoft Kinect and a desktop computer, without any additional add-ons or sensors for seniors to wear. Simple hardware setup and free from attachments can make seniors feel less complicated and cumbersome (54), leading to better convenience and usability. Thanks to the full-body motion tracking capabilities of Microsoft Kinect, older users can freely use their body gestures to directly interact with our system and perform intervention programs with joyful elements (9, 42). Our system software was carefully developed to seamlessly integrate multifactorial fall risk assessment and tailored interactive interventions so even older users were able to understand the workflow correctly. The system was tested beforehand to ensure clear instructions, no major malfunctions, and short loading times (53). In addition, system user interface was designed to be elder-friendly because the complexity of elements on-screen is strongly related to the bad impression of older people (55). We avoided putting too many complicated elements on the screen and only essential elements were displayed. The font or object size for key elements was determined to make it possible for at least 95% of the older population in Korea to read and recognize them (56).

It is worth noting that the older participants in this study had very limited experience with computers, as their overall mean CLS score was 2.5 out of 26. Nevertheless, the excellent SUS score of our system demonstrates that the developed Kinect-based tailored interactive fall intervention system was easy to use for older people, regardless of their computer experience. The 10th item of SUS (“I needed to learn a lot of things before I could get going with this system”) in our study had a relatively worse score than the other items (Figure 6). This can be related to the computer skills of our participants. They were diffident at operating the system totally independently because of their insufficient computer skills, even if they felt that the system was easy to use. Because of this, even after initial training, older participants can gradually and independently perform Kinect-based fall risk assessment and intervention programs by themselves, they still had difficulties in operating the entire system alone, especially when transitioning between different functions and intervention programs. On-site technical support was available upon request throughout the 8-week intervention. Therefore, our system usability may be somewhat overestimated and needs to be further improved.

This study has the following limitations. First, the study design was not a randomized controlled trial and the sample size was relatively small, therefore, the study findings may not be conclusive but only indicative. Future randomized controlled trials with larger sample sizes are needed to confirm the system effectiveness. Second, only community-dwelling older women participated in the experiment; therefore, it is not possible to determine whether the newly developed Kinect-based tailored interactive fall intervention system will be effective in other populations such as community-dwelling older men and patients with Parkinson disease (57). Third, no follow-up measurement was conducted. Even though the 8-week tailored intervention had positive effects on balance, functional mobility, cognitive function, and fear of falling, it is uncertain whether the improved status will be maintained after discontinuing the intervention programs. Last but not least, 6-month or 1-year prospective falls in both groups should be further collected to directly examine the effectiveness of the developed fall intervention system in reducing the risk of falls.

## Conclusion

In conclusion, this preliminary study examined the effectiveness and usability of a newly developed Kinect-based tailored interactive fall intervention system to reduce fall risk in older people. After an 8-week intervention, older participants' performance on TUG, BBS, and MoCA significantly improved, demonstrating the system's effectiveness in improving older people's physical and cognitive abilities. In addition, the system was easy to use for older people, regardless of their computer experience. Our findings suggest that the Kinect-based tailored fall intervention system could help health professionals and older people to proactively manage the risk of falls.

## Data Availability Statement

The raw data supporting the conclusions of this article will be made available by the authors, without undue reservation.

## Ethics Statement

The studies involving human participants were reviewed and approved by KAIST Institutional Review Board (IRB No: KH2021-194). The patients/participants provided their written informed consent to participate in this study.

## Author Contributions

TK designed experiment, acquired experimental data, performed statistical analysis, and drafted the manuscript. SX conceived and designed the study, obtained the funding, and reviewed and edited the manuscript draft. All authors reviewed and approved the final manuscript. All authors contributed to the article and approved the submitted version.

## Funding

This work was supported by the Basic Science Research Program through the National Research Foundation of Korea (NRF2017R1C1B2006811) and the ICT R&D innovative voucher support program through the Institute for Information & communications Technology Promotion (IITP20210019800012003).

## Conflict of Interest

The authors declare that the research was conducted in the absence of any commercial or financial relationships that could be construed as a potential conflict of interest.

## Publisher's Note

All claims expressed in this article are solely those of the authors and do not necessarily represent those of their affiliated organizations, or those of the publisher, the editors and the reviewers. Any product that may be evaluated in this article, or claim that may be made by its manufacturer, is not guaranteed or endorsed by the publisher.
